# Supplementary material for: Maraviroc inhibits SARS-CoV-2 multiplication and s-protein mediated cell fusion in cell culture
Source: bioRxiv. 2020 Aug 13:2020.08.12.246389. Preprint. [Version 1] doi: 10.1101/2020.08.12.246389 (PMC7430595; doi:10.1101/2020.08.12.246389)
Supplement: Supplementary file 1 [file NIHPP2020.08.12.246389-supplement-1.pdf]

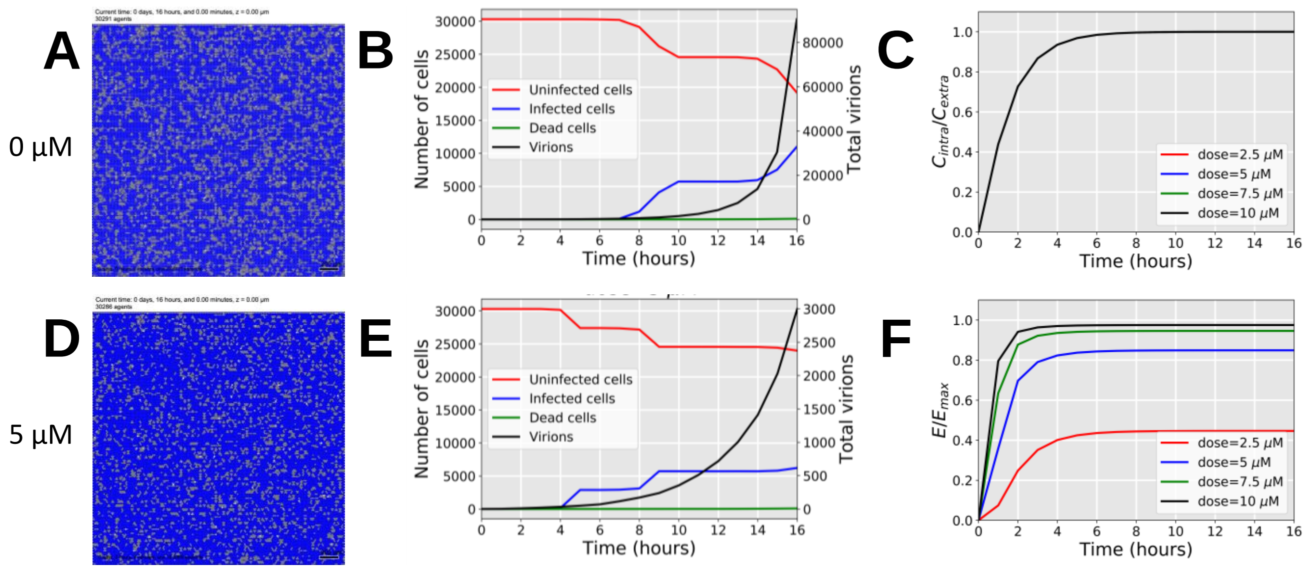

Fig S1: Calibrated model at 16 hours in 0 and 5 μM Maraviroc conditions. In untreated conditions (A-B), approximately 1/3 of the cells are infected, and many cells have reached high viral loads (yellow cells in A). When treated with 5 μM with Maraviroc, the reduced viral endocytosis and exocytosis leads to a reduction in the number of infected cells at 16 hours (D-E). Continued viral endocytosis and exocytosis is not likely due to drug absorption delays, as internalized drug (C) and drug effect (F) reach their maximal values within the first few hours.

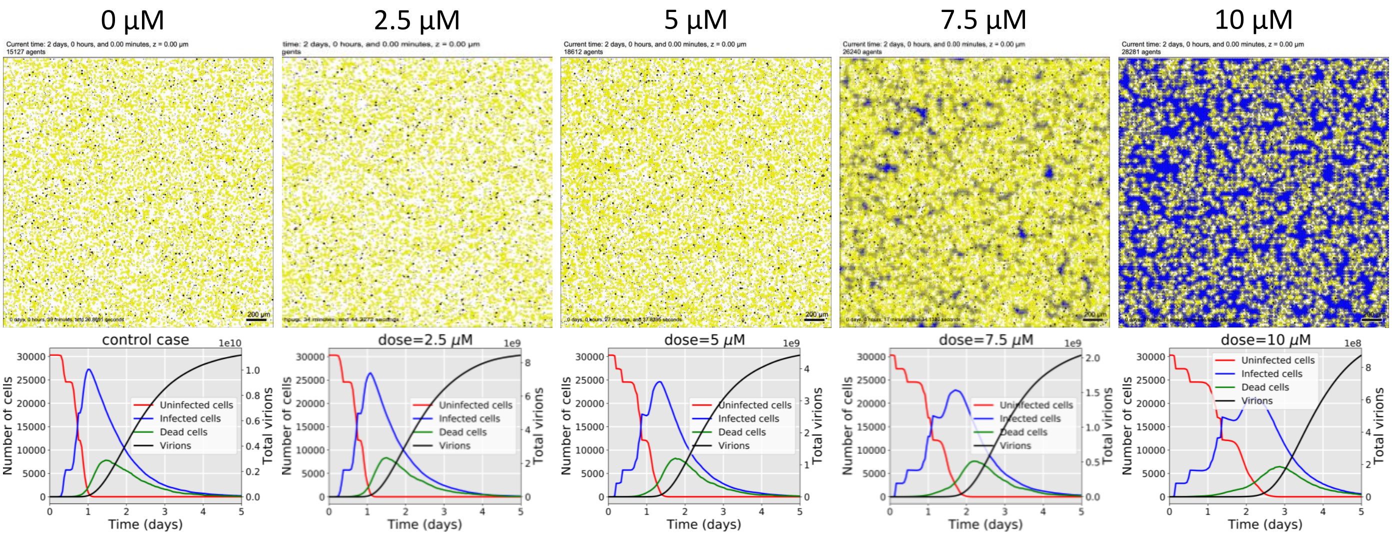

Fig S2: Impact of increasing the Maraviroc dose. *Top row:* As the dose is increased from 0 μM to 10 μM, the number of uninfected cells (deep blue) at 48 hours increases, while the number of infected cells with high viral load (bright yellow cells) and dead cells (black) decreases. *Bottom row:* As the dose is increased, the infection's progression is slowed, as seen by delayed increases in viral titer, infected cells, and dead cells. The final viral titer steadily decreases with increasing dose.

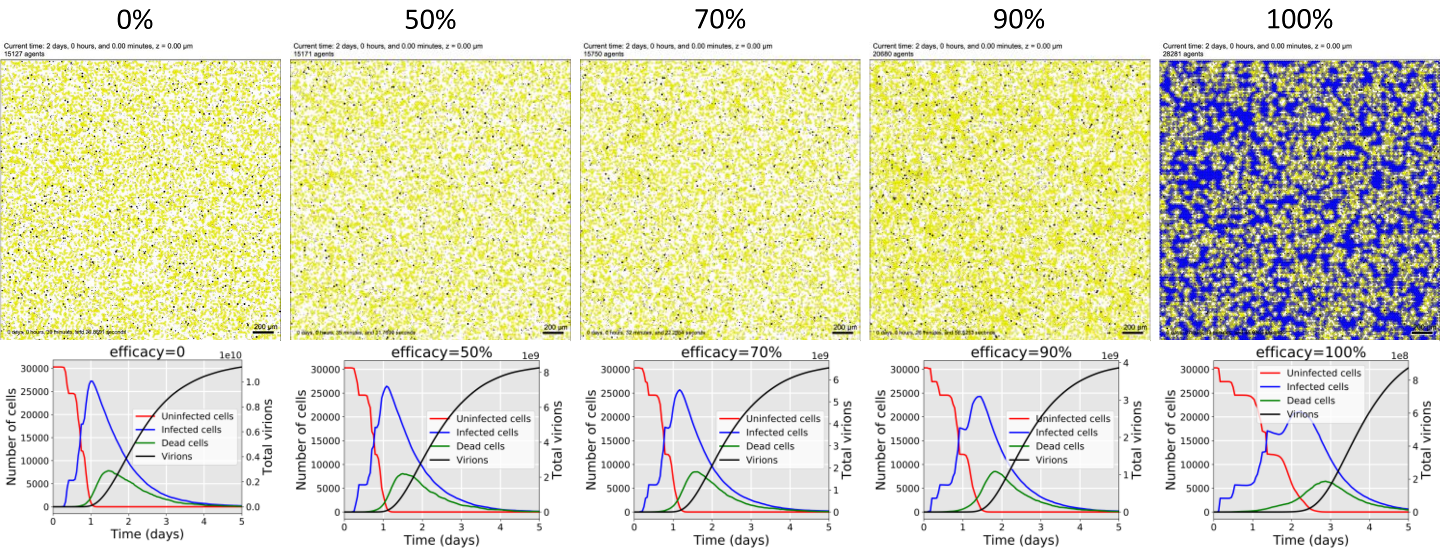

Fig S3: Impact of decreasing Maraviroc sensitivity. *Top row:* As the cells' maximum inhibitory response to a 10 μM dose of Maraviroc is increased from 0% inhibition of endocytosis and exocytosis to 100% inhibition, the number of uninfected cells (deep blue) at 48 hours increases, while the number of infected cells with high viral load (bright yellow cells) and dead cells (black) decreases. *Bottom row:* As sensitivity is increased, the infection's progression is slowed, as seen by delayed increases in viral titer, infected cells, and dead cells. The final viral titer steadily decreases with increasing dose.

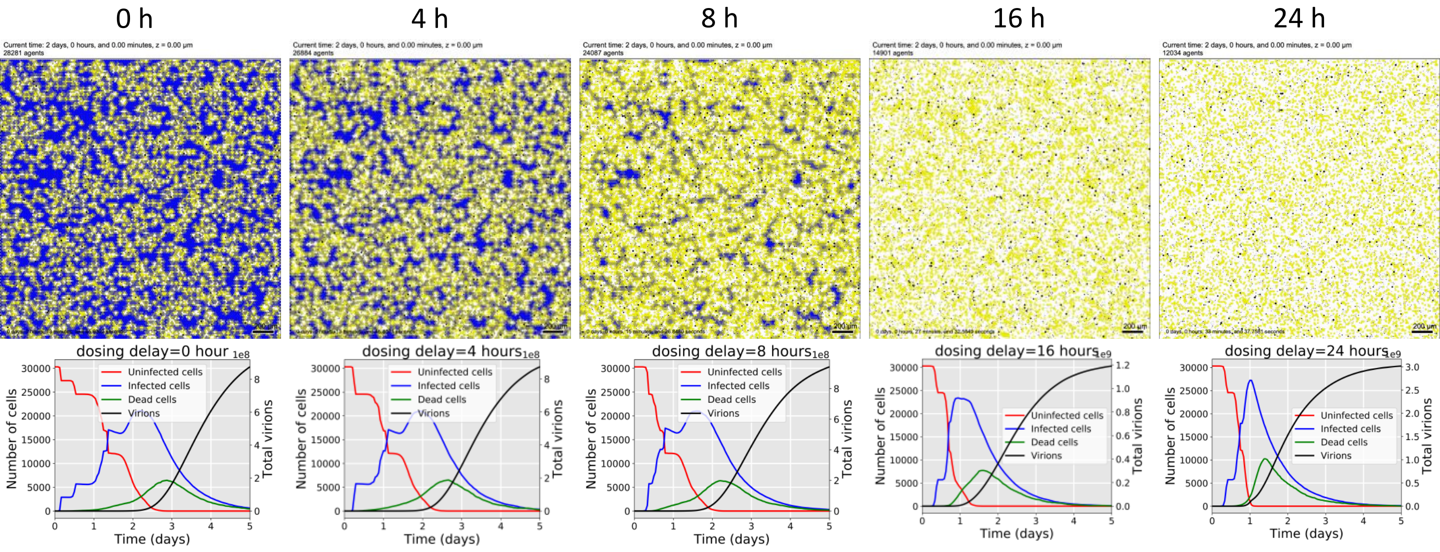

Fig S4: Impact of delaying Maraviroc treatment. *Top row:* As the 10 μM Maraviroc treatment is delayed by longer times, the number of uninfected cells (deep blue) at 48 hours increases, while the number of infected cells with high viral load (bright yellow cells) and dead cells (black) decreases. *Bottom row:* As the treatment delay is increased, the infection's progression is accelerated, as seen by earlier increases in viral titer, infected cells, and dead cells. The final viral titer steadily increases with increasing treatment delay.
